# Long-term medical and surgical treatment patterns in Korean patients newly diagnosed with primary open-angle glaucoma: A 10-year retrospective cohort study

**DOI:** 10.1371/journal.pone.0354241

**Published:** 2026-07-17

**Authors:** Sung Pyo Park, Chan Woong Joo, Kyoung Lae Kim, Youn Joo Choi, Kyeong Ik Na

**Affiliations:** Department of Ophthalmology, Kangdong Sacred Heart Hospital, Hallym University College of Medicine, Seoul, Korea; Brigham and Women’s Hospital, UNITED STATES OF AMERICA

## Abstract

**Purpose:**

To investigate the long-term medical and surgical treatment of patients with primary open-angle glaucoma (POAG).

**Methods:**

This retrospective cohort study analyzed data from the Health Insurance Review and Assessment Service (HIRA) database in Korea, focusing on patients newly diagnosed with POAG in 2010. The study examined glaucoma eye drop prescriptions and the occurrence of glaucoma surgery over a 10-year period, from 2010 to 2020. The main outcome measures included changes in glaucoma eye drop prescriptions and the cumulative incidence of glaucoma surgery following the initial diagnosis of POAG.

**Results:**

A total of 1,599 patients with POAG were enrolled. Prostaglandin analogue eye drops monotherapy was the most frequently prescribed glaucoma eye drop prescriptions in the first year (39.9%). The average number of prescribed glaucoma eye drops per patient was 1.19 in the first year and significantly increased to 1.40 by the tenth year. Similarly, the average number of prescribed ingredients per patient increased from 1.54 to 1.99 over the same period. The cumulative incidence of glaucoma surgery over 10 years was 3.1% (2.9% in males and 3.4% in females).

**Conclusions:**

This study quantitatively confirmed an increase in glaucoma medication use in patients newly diagnosed with POAG over a 10-year treatment period. Additionally, it reveals the incidence of glaucoma surgery based on sex and age. These findings provide valuable evidence for consulting patients with glaucoma.

## Introduction

Glaucoma is a chronic disease that requires long-term management to prevent progressive loss of vision. While primary glaucoma treatment involves the use of eye drops to lower intraocular pressure, surgical interventions may be necessary for some patients to achieve adequate control.

Patients newly diagnosed with glaucoma often experience significant anxiety and have many questions about disease progression and treatment course. Although extensive research has been conducted on the progression of glaucoma, particularly regarding retinal nerve fiber layer thinning [1–5] and visual field deterioration [6–10], relatively little attention has been paid to the long-term treatment trajectories that patients experience after diagnosis.

A limited number of studies have investigated glaucoma treatment patterns in real-world settings. Schwartz et al. analyzed treatment modifications among newly diagnosed open-angle glaucoma patients using a large United States administrative claims database and demonstrated that a substantial proportion of patients required treatment changes during a 4-year follow-up period [11]. More recently, Chuang et al. evaluated multinational trends in first-line glaucoma treatment between 2013 and 2024, providing important insights into evolving treatment preferences [12]. However, these studies primarily focused on short- to intermediate-term treatment modifications or changes in initial treatment selection. Consequently, long-term nationwide evidence describing how glaucoma treatment evolves after diagnosis, including both medical escalation and surgical intervention, remains scarce.

Providing patients newly diagnosed with glaucoma with clear information about their typical treatment course can help alleviate their fears and enable them to adhere more diligently to their treatment plans.

In this study, we analyzed the long-term treatment patterns of patients newly diagnosed with primary open-angle glaucoma (POAG) using data from the Health Insurance Review and Assessment (HIRA). Our aim was to quantify how medical treatment typically changes over time and determine the percentage of patients who ultimately underwent surgical intervention.

## Methods

This study was approved by the Institutional Review Board of [Name of Institution Withheld for Review] and complied with the tenets of the Declaration of Helsinki. Informed consent was not required.

### Data source

Approximately 53 million people in Korea are enrolled in compulsory health insurance programs. Specifically, 97.1% of the population is covered by the National Health Insurance Service, whereas the remaining 2.9% was covered by the Medical Aid Program or healthcare benefits for veterans. All the medical data for these insurance policies, including diagnoses, procedures, and prescription information, are stored in the HIRA database. Diagnoses in the HIRA database are coded using the Korean Standard Classification of Diseases, Version 5 (KCD 5), which is modified from the International Statistical Classification of Diseases and Related Health Problems, Tenth Revision (ICD-10).

We conducted a retrospective cohort study using data from the HIRA database collected between 2007 and 2020. Data were accessed for research purposes between August 1, 2022 and July 31, 2023. All data obtained from the HIRA database were de-identified, and the authors had no access to information that could identify individual participants during or after data collection.

### Participants

The data of patients with H40 code (a diagnosis of glaucoma) in 2010 were extracted from the HIRA database. To exclude patients who were previously diagnosed with glaucoma, those with H40 codes in 2007, 2008, and 2009 were excluded. Among patients who were first diagnosed with glaucoma in 2010, those who received only the H40.1 code (POAG) from 2010 to 2020 were included and those who received any of the following glaucoma diagnosis codes at least once were excluded: H40.2 (primary angle-closure glaucoma), H40.3 (glaucoma secondary to eye trauma), H40.4 (glaucoma secondary to eye inflammation), H40.5 (glaucoma secondary to other eye disorders), H40.6 (glaucoma secondary to drugs), H40.8 (other glaucoma), H40.9 (glaucoma, unspecified), H42.0 (glaucoma in diseases classified elsewhere), and Q15.0 (congenital glaucoma). Among them, we analyzed only those who were prescribed with glaucoma medication or underwent glaucoma surgery within the first year following their initial POAG diagnosis and had more than one year of follow-up after the diagnosis. The participants were divided into five age groups in 20-year intervals as follows: 0–19 years, 20–39 years, 40–59 years, 60–79 years, and ≥80 years.

### Medical glaucoma treatment

Prescribed glaucoma eye drops were classified as follows based on their active ingredients: alpha agonist eye drops (A), beta blocker eye drops (B), carbonic anhydrase inhibitor eye drops (C), pilocarpine eye drops (M), prostaglandin analogue eye drops (P), alpha agonist/beta blocker fixed-combination eye drops (AB), alpha agonist/carbonic anhydrase inhibitor fixed-combination eye drops (AC), carbonic anhydrase inhibitor/beta blocker fixed-combination eye drops (CB), and prostaglandin analogue/beta blocker fixed-combination eye drops (PB). Theoretically, 511 types of glaucoma eye drop prescription are possible.

We divided the study period for each participant into one-year intervals, starting from the day of their initial glaucoma diagnosis and spanning a total of 10 years. We designated the most frequently prescribed types of glaucoma eye drop prescription for each one-year period as the medical glaucoma treatment for that year for each participant.

### Surgical glaucoma treatment

We investigated whether the following six glaucoma surgery codes were registered for each participant over a 10-year period after their initial glaucoma diagnosis: S5033 (trabeculectomy), S5049 (glaucoma implant surgery), S5039 (iStent), S3038 (XEN), S5044 (cyclophotocoagulation), and S5054 (laser trabeculoplasty). In cases where the glaucoma surgery code was registered, we calculated the date from the initial glaucoma diagnosis to surgery and analyzed it on a yearly basis. Patients who underwent glaucoma surgery were considered to have missing values in the medical glaucoma treatment analysis after the date of glaucoma surgery.

### Statistical analysis

All data access and statistical analyses were conducted using RStudio version 1.1.463 (RStudio, PBC) through the HIRA’s remote access statistical analysis system.

We analyzed medical glaucoma treatments, focusing on the types of glaucoma eye drop prescriptions, the number of prescribed eye drops per patient, and the number of prescribed active ingredients per patient. Next, we conducted a linear regression analysis to examine the changes in the proportions over a 10-year period. We analyzed surgical glaucoma treatment by calculating the cumulative incidence of glaucoma surgery using the Kaplan–Meier survival estimator. We also used Cox regression to analyze adjusted hazard ratios (aHR) according to sex and age. Statistical significance was set to p < 0.05.

## Results

### Participants

Data from 2,144,203 patients with the H40 code in 2010 were extracted from the HIRA database. Among them, 1,004,170 patients with H40 codes from 2007, 2008, and 2009 were excluded. Of the 1,140,033 patients who were first diagnosed with glaucoma in 2010, we extracted 17,243 individuals who received a diagnosis using only the H40.1 code from 2010 to 2020. Among them, 1,599 patients were prescribed glaucoma medication or underwent glaucoma surgery within the first year following the H40.1 diagnosis and had more than one year of follow-up after the diagnosis.

Out of 1,599 individuals, there were 807 males (50.5%) and 792 females (49.5%), with a baseline mean age of 64.8 ± 14.4 years. The numbers of participants in the 0–19 years, 20–39 years, 40–59 years, 60–79 years, and ≥80 years age groups were 6, 89, 427, 843, and 234, respectively. The mean follow-up duration was 6.37 ± 3.24 years.

### Medical glaucoma treatment

The most frequently prescribed type of glaucoma eye drop prescriptions in the first year was P monotherapy (39.9%). However, it significantly decreased over time, reaching 24.3% in the tenth year (−1.44%/year, p < 0.001) (Table 1). Both B monotherapy (−0.47%/year, p < 0.001) and A monotherapy (−0.32%/year, p < 0.001) also showed significant decreases in prescription rates. In contrast, prescriptions of P + CB (0.99%/ year, p = 0.002), P + AB (0.33%/year, p < 0.001), and P + CB + A (0.64%/year, p < 0.001) showed significant increases over the same period.

**Table 1:** Glaucoma eye drop prescriptions for patients newly diagnosed with primary open-angle glaucoma over a 10-year treatment period.

	Follow up duration, number of patients (%)																			
Drug	1 year		2 year		3 year		4 year		5 year		6 year		7 year		8 year		9 year		10 year	
P	638	(39.9)	522	(33.0)	378	(31.4)	316	(30.8)	274	(29.9)	256	(30.3)	210	(27.5)	169	(24.2)	169	(25.6)	154	(24.3)
CB	202	(12.6)	247	(15.6)	180	(14.9)	155	(15.1)	139	(15.2)	133	(15.7)	109	(14.3)	107	(15.3)	101	(15.3)	91	(14.4)
B	134	(8.4)	99	(6.3)	69	(5.7)	53	(5.2)	48	(5.2)	34	(4.0)	32	(4.2)	25	(3.6)	23	(3.5)	23	(3.6)
AB	107	(6.7)	91	(5.8)	71	(5.9)	60	(5.8)	52	(5.7)	46	(5.4)	43	(5.6)	42	(6.0)	41	(6.2)	42	(6.6)
A	97	(6.1)	66	(4.2)	49	(4.1)	32	(3.1)	31	(3.4)	29	(3.4)	24	(3.1)	19	(2.7)	16	(2.4)	15	(2.4)
P + CB	82	(5.1)	154	(9.7)	146	(12.1)	142	(13.8)	140	(15.3)	126	(14.9)	127	(16.6)	109	(15.6)	102	(15.4)	102	(16.1)
C	77	(4.8)	6	(0.4)	4	(0.3)	3	(0.3)	3	(0.3)	3	(0.4)	5	(0.7)	0	0.0	1	(0.2)	2	(0.3)
PB	69	(4.3)	48	(3.0)	38	(3.2)	33	(3.2)	23	(2.5)	20	(2.4)	19	(2.5)	23	(3.3)	23	(3.5)	22	(3.5)
A + CB	32	(2.0)	34	(2.2)	33	(2.7)	29	(2.8)	23	(2.5)	16	(1.9)	14	(1.8)	18	(2.6)	14	(2.1)	15	(2.4)
P + C	26	(1.6)	7	(0.4)	7	(0.6)	6	(0.6)	5	(0.5)	6	(0.7)	7	(0.9)	9	(1.3)	5	(0.8)	9	(1.4)
P + A	24	(1.5)	29	(1.8)	27	(2.2)	21	(2.0)	18	(2.0)	21	(2.5)	14	(1.8)	12	(1.7)	11	(1.7)	11	(1.7)
P + AB	23	(1.4)	40	(2.5)	35	(2.9)	33	(3.2)	33	(3.6)	37	(4.4)	34	(4.5)	36	(5.2)	31	(4.7)	26	(4.1)
P + B	20	(1.3)	16	(1.0)	9	(0.7)	13	(1.3)	9	(1.0)	8	(0.9)	10	(1.3)	10	(1.4)	6	(0.9)	6	(0.9)
P + CB + A	20	(1.3)	31	(2.0)	39	(3.2)	41	(4.0)	41	(4.5)	40	(4.7)	42	(5.5)	40	(5.7)	46	(7.0)	46	(7.3)
Others	48	(3.0)	41	(2.6)	29	(2.4)	27	(2.6)	18	(2.0)	24	(2.8)	31	(4.1)	28	(4.0)	25	(3.8)	27	(4.3)
No use	0	(0.0)	149	(9.4)	91	(7.6)	62	(6.0)	59	(6.4)	46	(5.4)	43	(5.6)	52	(7.4)	47	(7.1)	42	(6.6)
Total	1599	(100.0)	1580	(100.0)	1205	(100.0)	1026	(100.0)	916	(100.0)	845	(100.0)	764	(100.0)	699	(100.0)	661	(100.0)	633	(100.0)

P, prostaglandin analogue eye drops; CB, carbonic anhydrase inhibitor/beta blocker fixed-combination eye drops; B, beta blocker eye drops; AB, alpha agonist/beta blocker fixed-combination eye drops; A, alpha agonist eye drops; C, carbonic anhydrase inhibitor eye drops; PB, prostaglandin analogue/beta blocker fixed-combination eye drops.

Among the 638 patients initially prescribed P monotherapy, 308 (48.3%) showed no changes in their prescription by the last follow-up year, whereas 85 patients discontinued P monotherapy during the follow-up period and remained off it until the last follow-up year (Table 2). Among the 638 patients initially prescribed P monotherapy, the prescription was changed to P + CB, CB, PB, and P + AB for 44 (6.9%), 26 (4.1%), 11 (1.7%), and 11 (1.7%) patients, respectively, during the follow-up period. Additionally, 10 (1.6%) patients changed their prescription two times, from P monotherapy to P + CB and then to P + CB + AB.

**Table 2:** Changes in glaucoma eye drop prescriptions in patients newly diagnosed with primary open-angle glaucoma over a 10-year treatment period.

Initial prescription	First changed prescription	Second changed prescription	Number of patients (percentage)	
P	no change		308	(48.3)
P	no prescription		85	(13.3)
P	P + CB		44	(6.9)
P	CB		26	(4.1)
P	PB		11	(1.7)
P	P + AB		11	(1.7)
P	P + CB	P + CB + A	10	(1.6)
P	P + A		9	(1.4)
P	others		134	(21.0)
Total			638	(100)
CB	no change		93	(46.0)
CB	P + CB		24	(11.9)
CB	no prescription		24	(11.9)
CB	P		11	(5.4)
CB	AB		6	(3.0)
CB	P + CB	P + CB + A	4	(2.0)
CB	CB + A		4	(2.0)
CB	others		36	(17.8)
Total			202	(100.0)
B	no change		46	(34.3)
B	no prescription		28	(20.9)
B	P		13	(9.7)
B	CB		8	(6.0)
B	others		39	(29.1)
Total			134	100.0

P, prostaglandin analogue eye drops; CB, carbonic anhydrase inhibitor/beta blocker fixed-combination eye drops; PB, prostaglandin analogue/beta blocker fixed-combination eye drops; AB, alpha agonist/beta blocker fixed-combination eye drops; A, alpha agonist eye drops.

**Fig 1:**
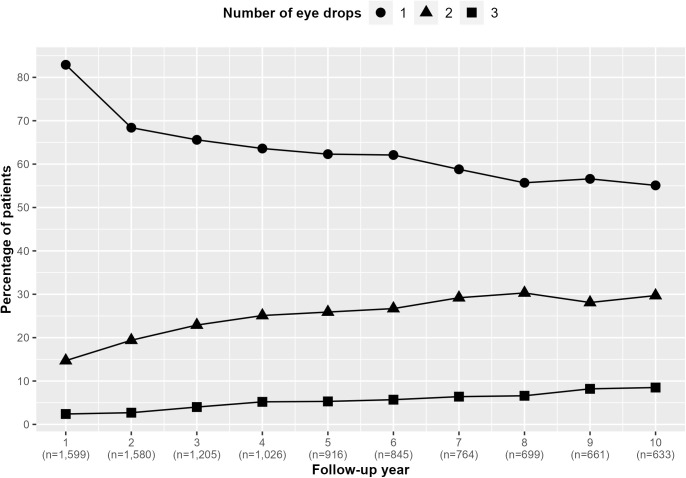
Percentage of patients based on the number of prescribed glaucoma eye drops per patient after the initial diagnosis of primary open-angle glaucoma.

The average number of prescribed glaucoma eye drops per patient was 1.19 in the first year and increased significantly in the following years (1.15, 1.23, 1.29, 1.30, 1.33, 1.36, 1.36, 1.37, and 1.40, respectively) (0.026/year, p < 0.001). The percentage of patients prescribed a single type of glaucoma eye drop was 82.9% in the first year. However, this percentage significantly decreased in the following years (68.4%, 65.6%, 63.6%, 62.3%, 62.1%, 58.8%, 55.7%, 56.6%, and 55.1%, respectively) (−2.41%/year, p < 0.001) (Fig 1). The percentage of patients prescribed two types of glaucoma eye drops was 14.7% in the first year and significantly increased in the following years (19.4%, 22.9%, 25.1%, 25.9%, 26.7%, 29.2%, 30.3%, 28.1%, and 29.7%, respectively) (1.49%/year, p < 0.001). The percentage of patients prescribed three types of glaucoma eye drops was 2.4% in the first year and significantly increased in the following years (2.7%, 4.0%, 5.2%, 5.3%, 5.7%, 6.4%, 6.6%, 8.2%, and 8.5%, respectively) (0.67%/year, p < 0.001).

**Fig 2:**
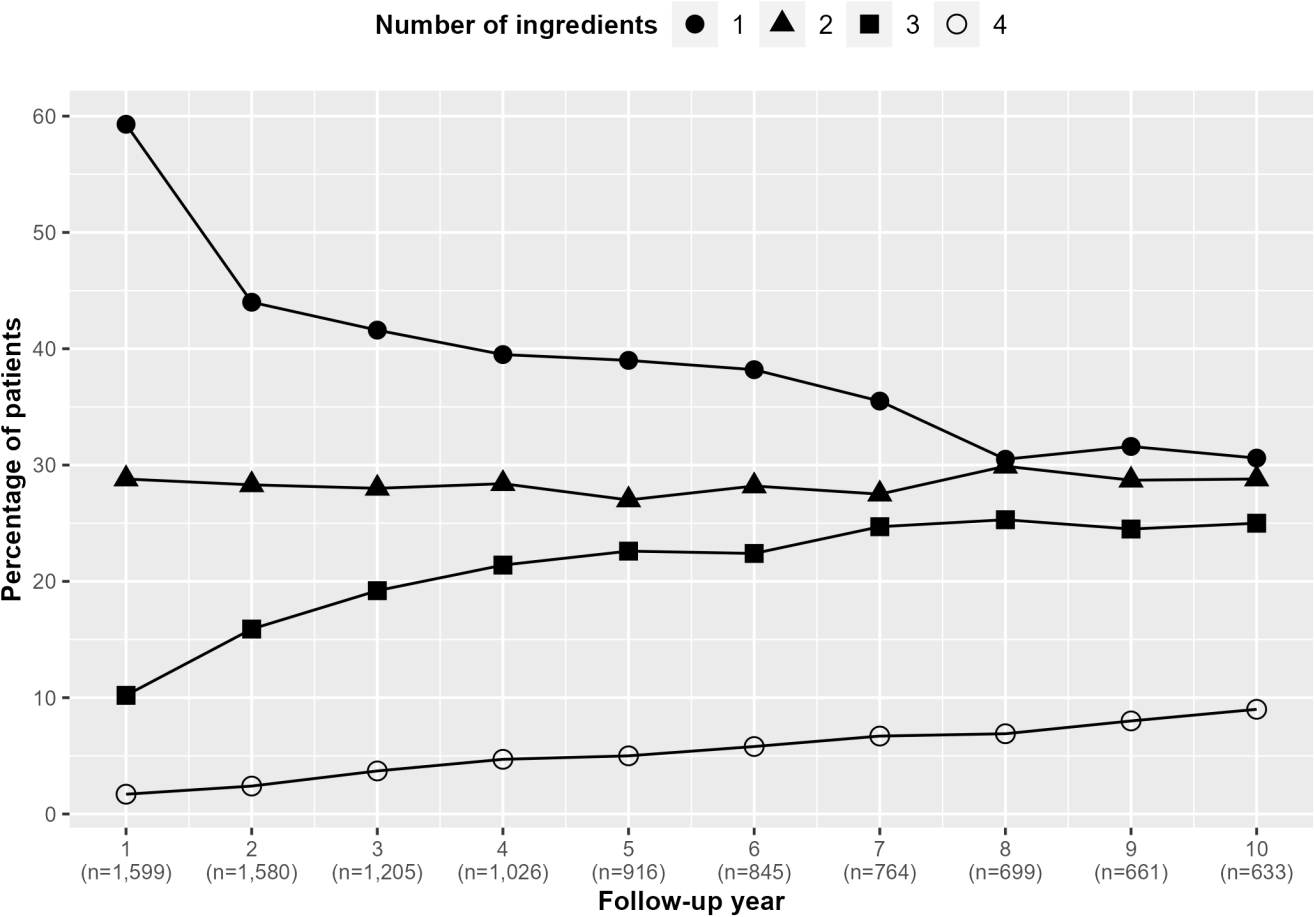
Percentage of patients based on the number of prescribed ingredients per patient after the initial diagnosis of primary open-angle glaucoma.

The average number of prescribed ingredients per patient was 1.54 in the first year and significantly increased in the following years (1.58, 1.70, 1.79, 1.81, 1.85, 1.91, 1.94, 1.95, and 1.99, respectively) (0.050/year, p < 0.001). The percentage of patients prescribed a single type of ingredient was 59.3% in the first year. However, this percentage significantly decreased in the following years (44.0%, 41.6%, 39.5%, 39.0%, 38.2%, 35.5%, 30.5%, 31.6%, and 30.6%, respectively) (−2.51%/year, p < 0.001) (Fig 2). The percentage of patients prescribed two types of ingredients was 28.8% in the first year and showed no significant change in the following years (28.3%, 28.0%, 28.4%, 27.0%, 28.2%, 27.5%, 29.9%, 28.7%, and 28.8%, respectively) (0.07%/year, p = 0.485). The percentage of patients prescribed three types of ingredients was 10.2% in the first year and significantly increased in the following years (15.9%, 19.2%, 21.4%, 22.6%, 22.4%, 24.7%, 25.3%, 24.5%, and 25.0%, respectively) (1.42%/year, p < 0.001). The percentage of patients prescribed four types of ingredients was 1.7% in the first year and significantly increased in the following years (2.4%, 3.7%, 4.7%, 5.0%, 5.8%, 6.7%, 6.9%, 8.0%, and 9.0%, respectively) (0.77%/year, p < 0.001).

### Surgical glaucoma treatment

**Fig 3:**
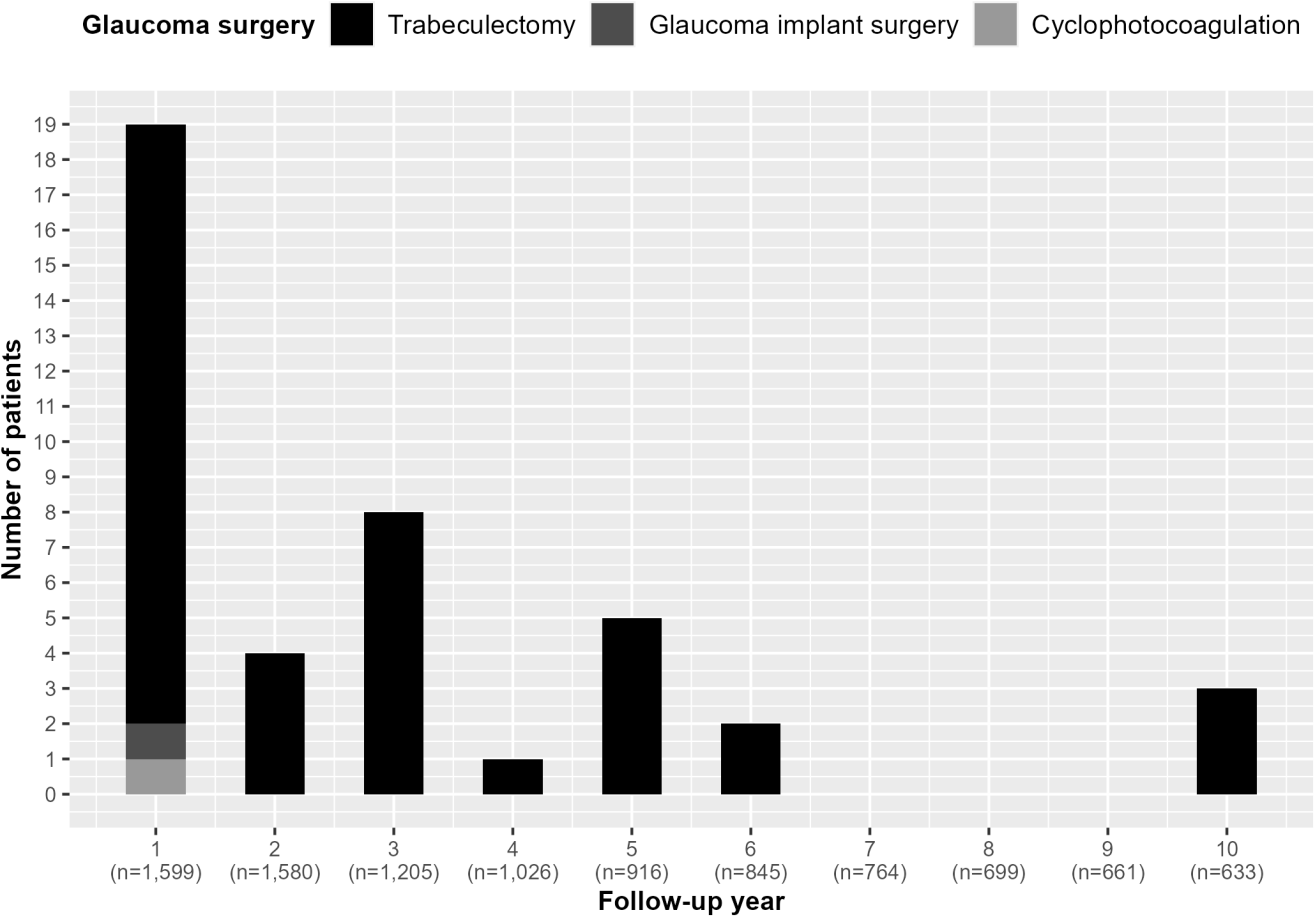
Number of patients who underwent glaucoma surgery after the initial diagnosis of primary open-angle glaucoma.

During the follow-up period, 42 (2.63%) patients underwent glaucoma surgery, 19 of whom underwent glaucoma surgery within the first year following the H40.1 diagnosis (Fig 3). Except for one patient who underwent glaucoma implant surgery and one patient who underwent cyclophotocoagulation, all other patients underwent trabeculectomy. The baseline mean age of the 42 patients who underwent glaucoma surgery was 57.8 ± 15.19 years, with 19 males and 23 females.

**Fig 4:**
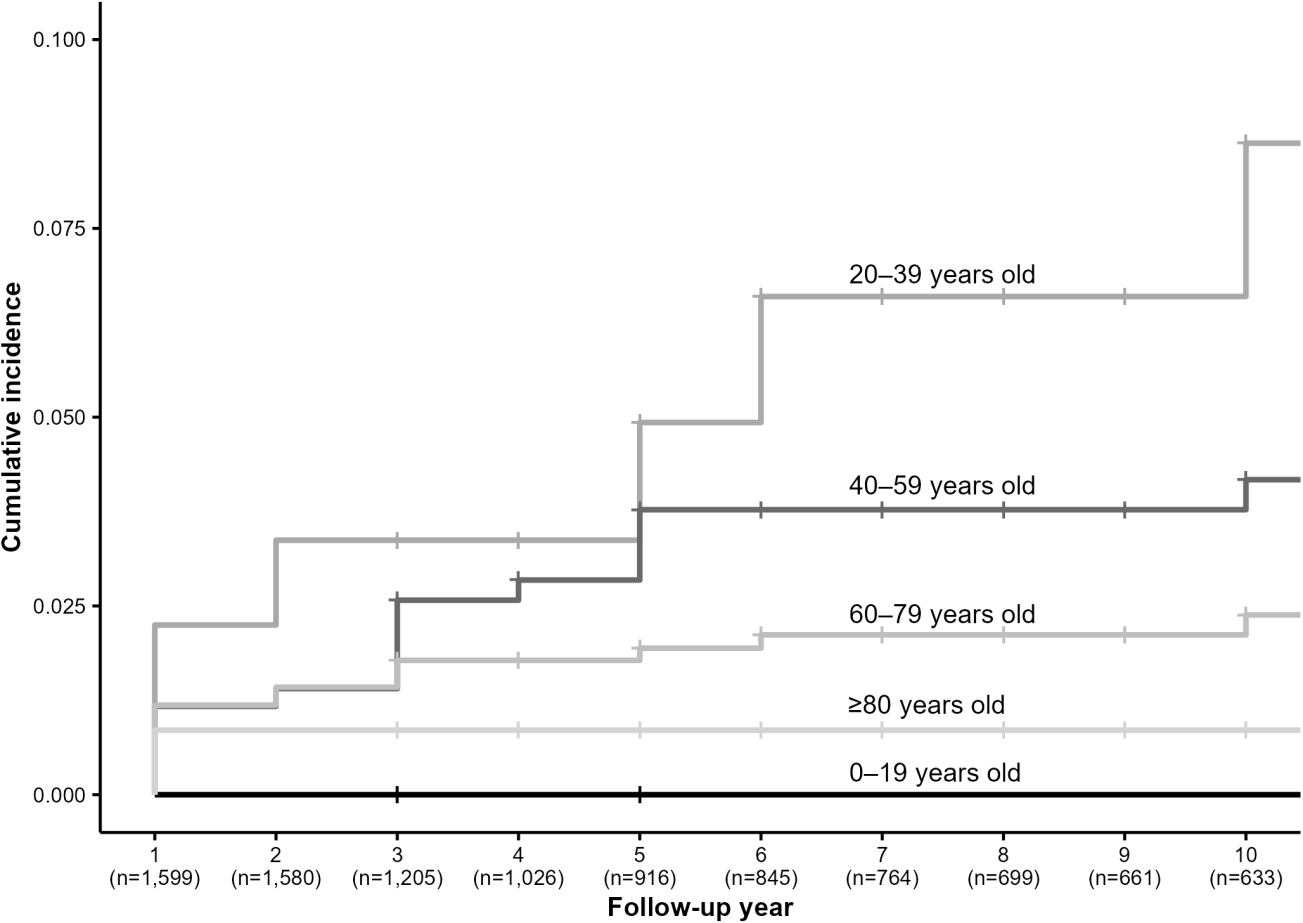
Cumulative incidence of glaucoma surgery in patients with newly diagnosed primary open-angle glaucoma stratified by age group over a 10-year treatment period.

The cumulative incidence of glaucoma surgery was 3.1% over 10 years using the Kaplan–Meier survival estimator. The cumulative incidence, stratified by age groups, was 8.6% for the 20–39 years age group, 4.2% for the 40–59 years age group, 2.4% for the 60–79 years age group, and 0.9% for the ≥ 80 years age group (Fig 4). None of the patients in the 0–19 years age group underwent glaucoma surgery within 10 years. The 20–39 years age group showed significantly higher risk of undergoing glaucoma surgery compared with the 60–79 years age group (aHR, 3.39 [95% confidence interval (CI), 1.34–8.58]; p = 0.010) and the ≥ 80 years age group (aHR, 7.46 [95% CI, 1.42–39.10]; p = 0.017) in multivariate Cox regression. The cumulative incidences of glaucoma surgery were 2.9% for male and 3.4% for female. Female sex was not significantly associated with glaucoma surgery (aHR, 1.43 [95% CI, 0.7739–2.65]; p = 0.253).

## Discussion

In this study, we analyzed the treatment journey of 1,599 patients newly diagnosed with POAG over a 10-year period using data from the HIRA. The average number of prescribed glaucoma eye drops per patient increased significantly from 1.19 to 1.40, and the average number of prescribed ingredients per patient increased significantly from 1.54 to 1.99. The cumulative incidence of glaucoma surgery over the 10-year period was 3.1%.

From a clinical perspective, these composite data offer a long-term ‘roadmap’ for managing newly diagnosed POAG patients. By understanding the typical progression of medication escalation and the 10-year cumulative incidence of surgery, clinicians can provide more personalized and evidence-based counseling. Sharing these long-term trends helps patients understand the chronic and progressive nature of glaucoma, which may enhance treatment adherence and prepare them for the possibility of future therapeutic adjustments.

In this study, we identified 17,243 patients who were first diagnosed with POAG in 2010 and subsequently received POAG diagnostic codes. This study relied solely on diagnostic and prescription codes as we were unable to access the patients’ actual charts. Additionally, there is a limitation in that a distinction between the right and left eyes is not made. To mitigate these limitations and obtain more precise data, we implemented strict criteria by excluding participants with glaucoma diagnostic codes other than H40.1. For the same reason, out of the 17,243 individuals, only 1,599 patients who were prescribed glaucoma medication or underwent glaucoma surgery within the first year following H40.1 diagnosis and had more than one year of follow-up after the diagnosis were included in the study.

P monotherapy was prescribed as the first glaucoma medication in 638 of 1,599 patients (39.9%). Prostaglandin analogues reduce intraocular pressure by increasing uveoscleral outflow [13,14]. Due to their efficacy, convenience of once-daily dosing, and safety profile, they are usually used as the first-choice therapy for POAG [11,15].

Among the 638 patients initially prescribed P monotherapy, 85 (13.3%) discontinued P monotherapy. Since we cannot review their charts to determine the exact reasons, we can speculate on the following situation. First, it could be a case where the patient initially received a prescription for glaucoma medication but discontinued it due to side effects or other reasons, opting instead to observe without using glaucoma medication. Second, although the diagnosis remained the same, there could have been a different assessment of the necessity for glaucoma medication. For example, a patient diagnosed with POAG and prescribed P by an ophthalmologist might seek a second opinion from another ophthalmologist and decide to observe the condition without using any glaucoma medication with the same POAG diagnosis.

The changes in glaucoma medication prescriptions after P monotherapy were diverse. Some patients switched from P to CB, where the ingredients of their glaucoma medication completely changed. Others transitioned from P to PB, shifting from single-component to fixed-combination glaucoma eye drops. There were also cases where medications were augmented, such as transitions from P to P + CB, P + AB, and P + A, in which additional glaucoma eye drops were added. Furthermore, some of the cases that switched to P + CB later had A added, resulting in P + CB + A.

During the follow-up of patients with glaucoma, the use of glaucoma medications is likely to increase owing to the progression of glaucoma. The results of the present study support this hypothesis. During the first year of follow-up, an average of 1.19 glaucoma eye drops per patient and 1.54 ingredients per patient were prescribed. This indicates that 0.84 single-component glaucoma eye drops and 0.35 fixed-combination glaucoma eye drops were prescribed. In the final year of follow-up, an average of 1.40 glaucoma eye drops per patient and 1.99 ingredients per patient were prescribed. This indicates that 0.81 single-component glaucoma eye drops and 0.59 fixed-combination glaucoma eye drops were prescribed. Based on the above results, the augmentation of glaucoma medications can be attributed to an increase in the use of fixed-combination glaucoma eye drops. It can be inferred that fixed-combination eye drops, with its advantages such as increased patient compliance and reduced side effects, are being considered for the treatment of glaucoma [16–23].

In this study, none of the patients in the 0–19 years age group underwent surgery during the study period. The 0–19 years age group comprised six individuals. If glaucoma was moderately present at the initial diagnosis within this age range, it was highly likely that the glaucoma was caused by congenital abnormalities; thus, patients were more likely to be diagnosed with congenital glaucoma rather than POAG. Therefore, it is thought that the 0–19 years age group included in this study primarily consisted of relatively older patients with lower severity of glaucoma, which likely resulted in a lower need for surgery. This is supported by the fact that the average age of the six individuals in the 0–19 years age group was 14.3 ± 3.1 years.

In contrast, the 20–39 years age group showed the highest cumulative incidence of glaucoma surgery, with the cumulative incidence decreasing sequentially in the 40–59 years, 60–79 years, and ≥80 years age groups. Age is one of the significant risk factors for POAG [24]. The younger the age at which POAG is diagnosed, the more likely it is that other risk factors such as high intraocular pressure, [25,26] myopia, [27–29] diabetes, [30] hypertension, [31] and genetic factor [32,33] have influenced the development of POAG rather than age itself. In these younger patients, the passage of time, as represented by aging, might accelerate the progression of glaucoma. Therefore, it can be inferred that although the incidence of POAG decreases with younger age, the likelihood of requiring glaucoma surgery during follow-up increases.

There are several limitations to this study that should be considered. First, this investigation is a retrospective cohort study using a national claims database, which makes it inherently vulnerable to various sources of bias. Second, the 1,599 patients analyzed represent only a portion of the total POAG population in Korea who met our specific inclusion criteria for a 10-year follow-up; this may introduce selection bias and limit the generalizability of our findings to the broader glaucoma population. Third, because the HIRA database is based on prescription records rather than actual medication intake, the actual adherence to medical treatment by individual patients remains unknown. Finally, because this study relied on a national health registry without a direct review of medical records, clinical parameters were not available to correlate treatment patterns with disease severity.

In this study, we analyzed the treatment journey of patients newly diagnosed with POAG using a database that included diagnosis and treatment codes for the entire population of South Korea. Most patients begin glaucoma treatment with prostaglandin analogue monotherapy, subsequently changing or increasing the dosage of their glaucoma eye drops in various ways. The average number of prescribed glaucoma eye drops and ingredients per patient has increased significantly. The cumulative incidence of glaucoma surgery tended to decrease with increasing age.
